# Efficient Percutaneous Delivery of the Antimelanogenic Agent Glabridin Using Cationic Amphiphilic Chitosan Micelles

**DOI:** 10.1371/journal.pone.0164061

**Published:** 2016-10-03

**Authors:** Haruyoshi Seino, Yukari Arai, Norio Nagao, Noriyasu Ozawa, Kazuhiko Hamada

**Affiliations:** 1 Central Research Laboratory, Pias Corporation, 1-3-1 Murotani, Nishi-ku, Kobe, Japan; 2 Faculty of Life and Environmental Science, Prefectural University of Hiroshima, 562 Nanatsuka, Shobara, Japan; University of Alabama at Birmingham, UNITED STATES

## Abstract

Partially myristoylated chitosan pyrrolidone carboxylate (PMCP) is a cationic amphiphilic chitosan derivative. Glabridin (Glab) from licorice root extracts is a hydrophobic antimelanogenic agent. Here we assessed the effects of cationic Glab-containing polymeric micelles derived from PMCP (Glab/PMCP-PM) on the ability of Glab to penetrate the skin and inhibit melanogenesis using a human skin model. The amount of Glab absorbed 24 h after the application of Glab/PMCP-PM was approximately four times higher than that of conventional oil-in-water micelles (control) prepared using Tween 60. Further, the release of IL-1α, a mediator of inflammation, was not detected. Treatment with Glab/PMCP-PM significantly increased the inhibition of melanogenesis compared with control. The inhibition of melanogenesis depends upon the enhanced ability of Glab to penetrate the skin, particularly the epidermis. Moreover, the inhibition of melanogenesis and the cationic potential of the Glab/PMCP-PM levels were increased by the cationic phospholipid copolymer. Therefore, Glab/PMCP-PM shows potential as an effective transdermal delivery system for treating skin hyperpigmentation.

## Introduction

Skin hyperpigmentation is caused by the overproduction of melanins, which are synthesized by tyrosinase and melanogenesis related proteins (MRP) [1]. Tyrosinase mediates melanogenesis, which frequently occurs in damaged human skin. It has reported that L-tyrosine and L-DOPA enhance tyrosinase and its transport into melanosomes results in an increase in melanin pigmentation [2,3]. Uneven pigmentation, particularly of facial skin, is often masked using make-up cosmetics. However, the treatment of injured skin is invariably an ongoing process that requires considerable effort [4]. Therefore, there is great demand for medicated skin care cosmetics and medications for external application that are clinically designed to remedy or prevent skin disorders.

Melanin synthesis inhibition agents, which are extensively used to treat hyperpigmentation, include a variety of antimelanogenic agents such as ascorbic acid derivatives [5], arbutin (4-hydroxyphenyl-β -D-glucopyranoside) [6,7], kojic acid (5-hydroxy-2-hydroxymethyl-4H-pyran-4-one) [8], and glabridin (3-(2′,4′-dihydroxyphenyl)-8-dimethyl-pyrano[8,7e]chroman) [9,10]. The mode of action of these agents is related to tyrosinase activity. Strategies to enhance the percutaneous delivery of these antimelanogenic agents often include using surfactants or alcohols. Unfortunately, these substances can disrupt the skin’s barrier function and induce a rash or irritation [11,12]. Thus, new transdermal delivery systems are required to selectively increase the permeability of the skin to antimelanogenic agents without compromising the skin’s barrier function.

**Fig 1:**
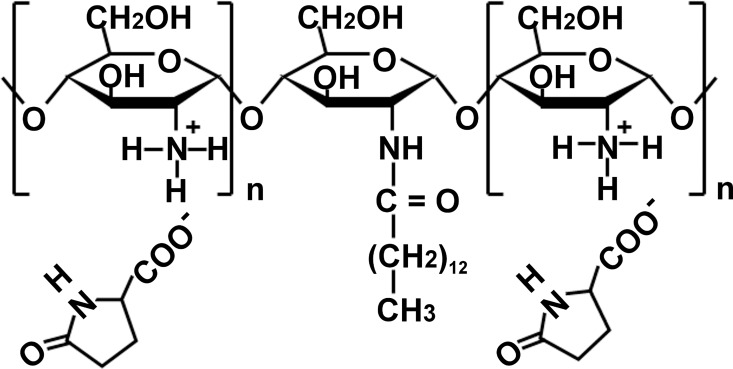
Structure of partially (10%) myristoylated chitosan pyrrolidone carboxylate (PMCP).

Chitosan is a natural polysaccharide that is rich in amino residues and possesses several interesting characteristics, including moisturizing effects [13] and bactericidal and wound-healing properties [14]. It was suggested that chitosan and partially acetylated chitosan are usable as digestible materials in the biomedical and biotechnological fields [15,16]. Chitosan with the amino group as its cationic sites is hydrophilic because of the protonation of its amino groups at an acidic pH [17,18]. However, myristoylation of the polymer increases its hydrophobicity [18]. Sulfated *N*-acyl-chitosan comprising long alkyl chains forms a novel “polymer micelle,” which is more stable than conventional micelles that are formed using low molecular weight surfactants [19]. We synthesized partially myristoylated (10%) chitosan pyrrolidone carboxylate (PMCP) **(Fig 1)** as a cationic amphiphilic chitosan [20,21]. PMCP is a novel oil-in-water (O/W) emulsifier that forms crosslinks between myristoyl (C_14_) groups that anchor fat-soluble substances. PMCP is a nontoxic and biodegradable cationic polymer. Because O/W emulsions derived from chitosan do not contain a low molecular weight surfactant, they are particularly useful in the manufacture of hypoallergenic cosmetics.

Glabridin (Glab) is a pyranoisoflavan isolated from licorice. Glab, the main compound in the hydrophobic fraction of licorice root extract, is a flavonoid and effective inhibitor of melanogenesis and inflammation [10]. To increase the skin permeability without irritation and suppress skin pigment synthesis, we prepared novel nanoparticle-sized cationic polymeric micelles enclosing Glab emulsified using PMCP. In this paper, these micelles were prepared using high-pressure homogenization. Human skin is negatively charged at physiological pH and permselective for cations [22]. Therefore, cationic micelles are suitable for mediating transdermal absorption. Here we used a three-dimensional model of the human skin to investigate the influence of these cationic polymeric micelles on the ability of Glab to penetrate the skin and inhibit melanogenesis.

## Materials and Methods

### Materials

**Fig 2:**
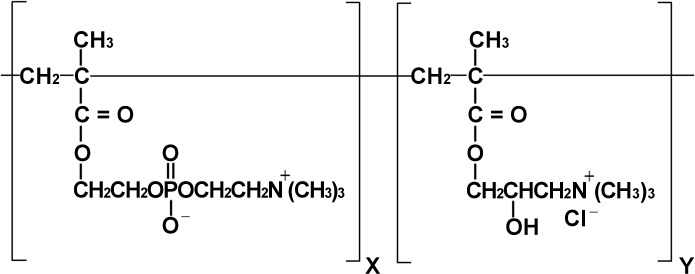
Structure of cationic phospholipid copolymer (PQ-64). X:Y = 8:2, where X represents 2-methacryloyloxyethyl phosphorylcholine and Y represents (2-hydroxy-3-methacryloyloxypropyl) trimethylammonium chloride.

PMCP (trade name PM-Chitosan) was supplied by Pias Co., Ltd. (Osaka, Japan). The cationic phospholipid copolymer polyquaternium-64 (PQ-64) (trade name Lipidure C) was purchased from NOF Corporation (Tokyo, Japan). PQ-64 comprises 2-methacryloyloxyethyl phosphorylcholine and (2-hydroxy-3-methacryloyloxypropyl) trimethylammonium chloride **(Fig 2)**. A 40% preparation of Glab from licorice root extract (Glab-40) was supplied by Maruzen Pharmaceuticals Co., Ltd. (Hiroshima, Japan) [10]. The anionic polymer–surfactant acrylic acid alkyl copolymer (AACP) (trade name Pemulen TR-1) was purchased from Nikko Chemicals Co., Ltd. (Tokyo, Japan). Polyoxyethylene sorbitan monostearate (Tween 60) was purchased from Kao Corporation (Tokyo, Japan). All other chemicals were reagent grade.

### Preparation of Glab-containing PMCP polymeric micelles (Glab/PMCP-PM)

Glab/PMCP-PM was prepared using a pressure homogenization method as follows: Polymeric emulsions comprising PMCP, Glab-40, PQ-64, butylene glycol, cetyl ethylhexanoate, and purified water were prepared by adding an aqueous phase to an oily phase and homogenized using a Homomixer (TK Homo Mixer, Primix, Osaka, Japan). Glab/PMCP-PM suspensions were prepared with two passages through a high-pressure homogenizer (Nanomizer NM; Yoshida Kikai Co., Ltd. Nagoya, Japan) at 20,000 psi. The concentrations of PMCP, Glab-40, PQ-64, butylene glycol, and cetyl ethylhexanoate in Glab/PMCP-PM suspensions were 0.2% (w/w), 0.015% or 0.06% (w/w), 0% or 0.1% (w/w), 5.0% (w/w), and 2.0% (w/w), respectively. However, Glab-containing O/W nanomicelles were prepared using 0.5% Tween 60 or 0.2% AACP using the same conditions as those used for preparing PMCP micelles.

### Characterization of polymeric micelles

The zeta potentials and average particle sizes of polymeric micelles or nanomicelles were determined by dynamic light-scattering analysis and laser diffraction spectrometry, respectively, using a Zetasizer Nano Series (Nano ZS90, Sysmex, Kobe, Japan). The morphology of the polymeric micelles was monitored using a transmission electron microscope (HITACHI H-7600, Hitachi Ltd, Tokyo, Japan) operating at 100 kV, and the samples were negatively stained with a 2.0% uranylacetate solution.

### Transdermal penetration assay for Glab using a human skin model

Reconstituted commercial skin models (TESTSKIN LSE-d or TESTSKIN LSE-high; Toyobo Co. Ltd., Osaka, Japan) comprising a multilayered skin structure that was formed by the growth of human keratinocytes on human dermal fibroblasts adhered to a collagen-coated filter. The skin cultures were placed in 12- or 6-well plastic culture dishes containing 1 or 1.5 ml per well of assay medium (supplied with the TESTSKIN culture kits) supplemented with one of the test polymeric micelle suspensions, which was applied to the tissue surface. The samples were incubated at 37°C for 24/48 h in a humidified atmosphere containing 5% CO_2_. Glab absorbed in the skin tissue was extracted as follows: After the specified incubation period, the surface of the skin model was rinsed 10 times with 1-ml phosphate-buffered saline (PBS) to remove surplus test sample. Skin cells were obtained by boring 10-mm diameter holes. Glab that was absorbed into the interior of the skin model was extracted as follows: Isolated tissues were transferred to Eppendorf tubes containing 1.0 ml of ethanol and then sonicated for 1 h. Glab concentration in the supernatant of the cell-free extract was measured using HPLC. The separation of Glab was performed using a TOSOH-GEL column (4.6 × 256 mm; Tosoh, Yamaguchi, Japan) at a column temperature of 40°C. The mobile phase was 2% acetic acid:acetonitrile (40:60) delivered at 1.0 ml min^−1^. The ultraviolet light absorbance of the eluent was monitored at 282 nm. The retention time of Glab was 9.8 min.

### Cell pigmentation assay in the human skin model containing functional melanocytes

A commercial three-dimensional human skin equivalent (MEL-300-A; MatTek Corp., Ashland, MA, U.S.A) was used for cell viability tests and pigment production assays [23, 24]. MEL-300-A comprises human-derived epidermal keratinocytes and melanocytes that form a multilayered and highly differentiated model of the human epidermis. The cells were cultured in 6-well plates, and the medium was supplemented using EPI-100-LLMM culture kits (Kurabo Industries, Osaka, Japan). Before treatment with test samples, the skin cultures were placed in wells containing EPI-100-LLMM assay medium (0.9 ml per well) and incubated for 1 h at 37°C in a humidified atmosphere containing 5% CO_2_. The cells were treated for 10 days, the medium was changed every second day, and then the surface of the tissue was rinsed five times with 1 ml of PBS to remove surplus test sample. The viability of the skin tissue was determined using the Alamar Blue assay [25]. The medium was removed. The human skin model was placed in a 24-well plate, and 0.3 ml of Alamar Blue solution (diluted 10-fold with EPI-100-LLMM) was added to each well and incubated for 1 h as described above. Sample absorbance was measured using an automatic microplate reader (SH-9000; Corona Electric Co., Ltd. Ibaraki, Japan) at 570 nm and 600 nm excitation and emission wavelengths, respectively. Skin tissue (90% viable) was fixed using 10% formalin (Sigma-Aldrich). Pigmentation of the skin tissue was assessed by the L* value-estimated skin brightness [23]. With respect to the L* parameter in the spectrophotometer, the L* value indicates lightness, the increase indicates more brightness, and the decrease indicates more darkness. Reflectance spectra of skin surface color and hue degree were measured by the spectrophotometer (Shimadzu UV-2450; Shimadzu Corp., Kyoto, Japan) with the integrating sphere attachment over every wavelength from 400 to 800 nm. The L* value was analyzed using optional color measurement software (COL-UVPC: Shimadzu Corp.) from the spectra obtained by this spectrophotometer. In addition, the skin tissue was cut at the mid-portion of the skin surface and each part was embedded in paraffin. Melanin pigments in the skin tissue section were stained using the Fontana-Masson method that is used to histochemically visualize melanin [26, 27, 28].

### Measurement of IL-1α release from the human skin model

The test sample was added to the reconstituted skin (TESTSKIN LSE-high) for 24 h, the medium was collected, and IL-1α was quantified using an ELISA (Biotrak Human Interleukin-1 alpha ELISA system; Amersham Biosciences Corp, USA).

### Statistical analysis

Differences between the mean values of groups were assessed using one-way AVONA with the Bonferroni’s post-test or unpaired Student’s *t*-test. The correlations were analyzed by a Pearson’s test. A p of < 0.05 was considered statistically significant.

## Results

### Properties of micelle forming by PMCP

**Fig 3:**
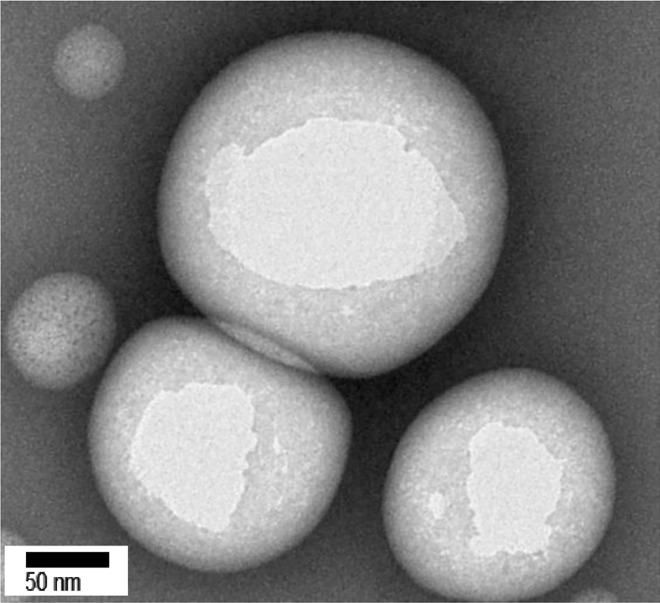
Morphology of PMCP polymeric micelles. Electron micrograph of negatively stained PMCP micelles.

We previously reported that PMCP is a cationic amphiphilic chitosan derivative [21]. The physical properties of cationic polymeric micelles derived from PMCP were examined. The average diameter and zeta potential of the spherical Glab/PMCP-PM particles were 188.0 ± 31.7 nm and 32.1 ± 0.9 mV, respectively **(Fig 3)**. The addition of PQ-64 to Glab/PMCP-PM increased the zeta potential to 57.9 ± 1.7 mV. However, no difference in average particle size (183.0 ± 21.2 nm) was observed compared with that of Glab/PMCP-PM, suggesting that the cationic potential of Glab/PMCP-PM was enhanced by PQ-64.

### Cationic chitosan micelles effect on the percutaneous transport of Glab

**Fig 4:**
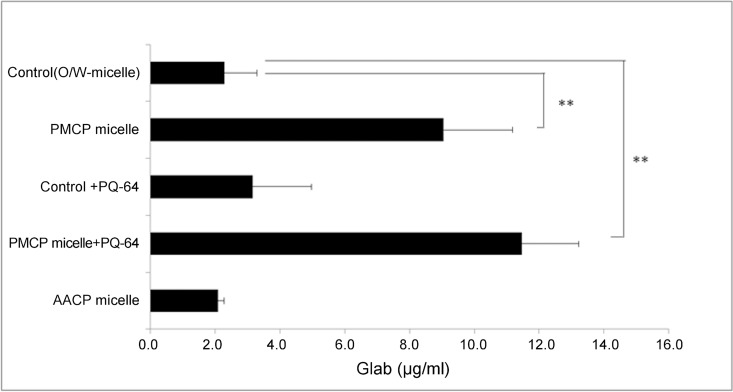
PMCP polymeric micelles enhanced the effects of Glab absorption. Glab-containing micelles were applied to a human skin model (TESTSKIN LSE-d). The amount of Glab absorption was measured 24 h after test sample application. Significant differences from control values are indicated (**p < 0.01, ANOVA).

To investigate the percutaneous delivery of Glab, the effect of Glab/PMCP-PM on the percutaneous transport of Glab was examined. The amount of Glab in skin after Glab/PMCP-PM application to the model was approximately four times higher than that delivered by conventional O/W nanomicelles (average particle size 152.0 ± 40.3 nm and zeta potential 6.8 ± 0.2 mV), which were formed using 0.5% of the nonionic low molecular weight surfactant Tween 60 **(Fig 4)**. Further, using the conventional O/W nanomicelle procedure, PQ-64 addition to Glab/PMCP-PM increased the percutaneous delivery of Glab but had no such effect on promoting the skin permeability of Glab. Note that the effect of PQ-64 on the percutaneous delivery of Glab to the skin model was restricted to polymeric PMCP micelles. No difference was observed in the amounts of Glab in skin tissues delivered using anionic polymer micelles (average particle size 205.0 ± 10.8 nm, zeta potential −17.4 ± 1.7 mV) formed using Pemulen TR-1 and conventional O/W micelles.

**Fig 5:**
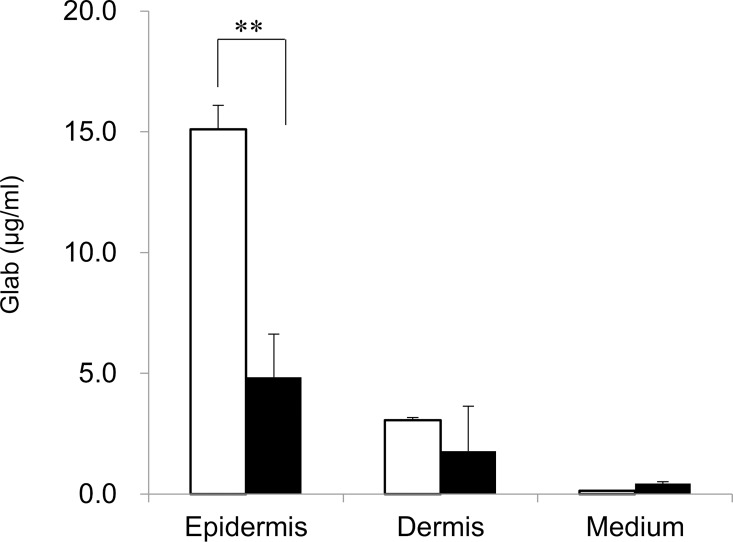
Distribution of Glab in a reconstituted human skin model. PMCP micelle (open bar) or O/W micelle (closed bar) was applied to the human skin model (TESTSKIN LSE-high). Distribution of Glab was measured 24 h after test sample application. Significant differences are indicated (** p < 0.01, *t*-test).

**Fig 6:**
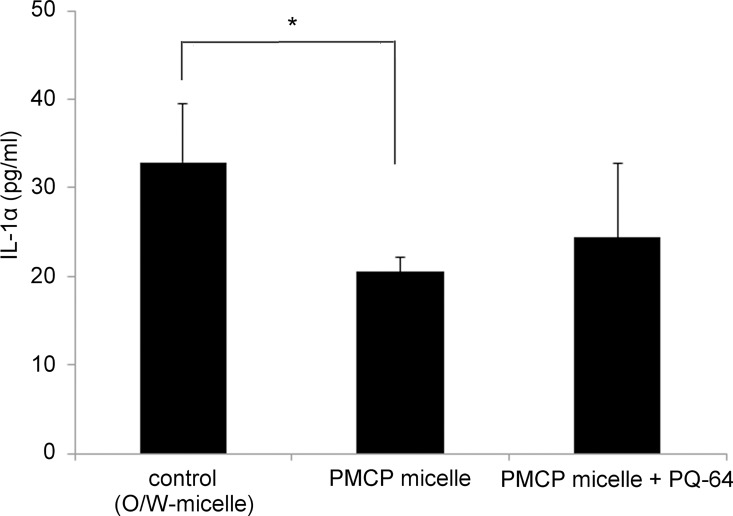
Release of IL-1α from TESTSKIN after Glab application. The amount of IL-1α in the medium was quantified using ELISA. Significant differences from control values are indicated (*p < 0.05, ANOVA).

We next assessed the effect of nanomicelles on the distribution of Glab in the human skin model. The distribution of Glab in the model (LSE-high) is shown in **Fig 5**. Glab was mainly observed in the epidermis when applied using Glab/PMCP-PM and reached a level that was approximately three times higher than that of conventional O/W micelles. There was no significant difference in the Glab levels in the dermis delivered by the two types of micelles and the amounts of Glab in the culture fluids, which were very low in both samples. Despite the increased penetration of Glab, the application of Glab/PMCP-PM or Glab/PMCP-PM containing PQ-64 had no effect on the stimulation of IL-1α levels compared with conventional treatment with Glab using O/W micelles **(Fig 6)**, which is consistent with our findings that PMCP does not affect cell viability and does not induce IL-1α production [21].

### Melanogenesis inhibition by Glab/PMCP-PM in the human skin model

**Fig 7:**
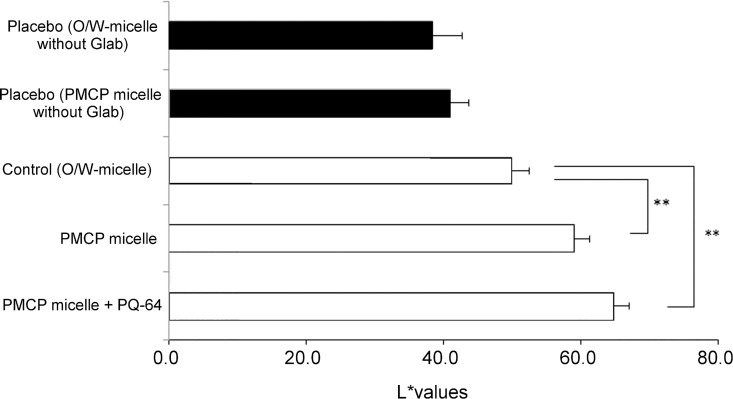
Inhibition of melanogenesis in a reconstituted human skin model by PMCP polymeric micelles containing Glab. Test sample was incubated with the human skin model (MEL-300-A) and incubated for 10 days. The L* value of the skin surface was assayed. Significant differences from control values are indicated (**p < 0.01, ANOVA).

**Fig 8:**
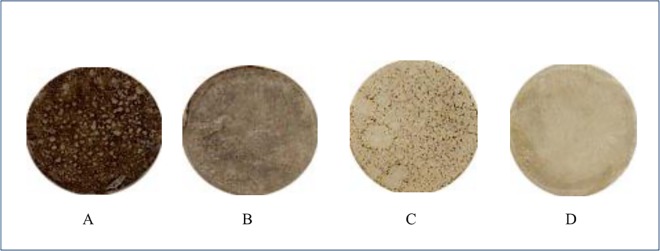
Macroscopic views of a reconstituted human skin model. The human skin model (MEL-300-A) was incubated as described in Fig 7. (A) control (O/W micelle without Glab); (B) O/W micelle; (C) PMCP micelle; (D) PMCP micelle + PQ-64.

**Fig 9:**
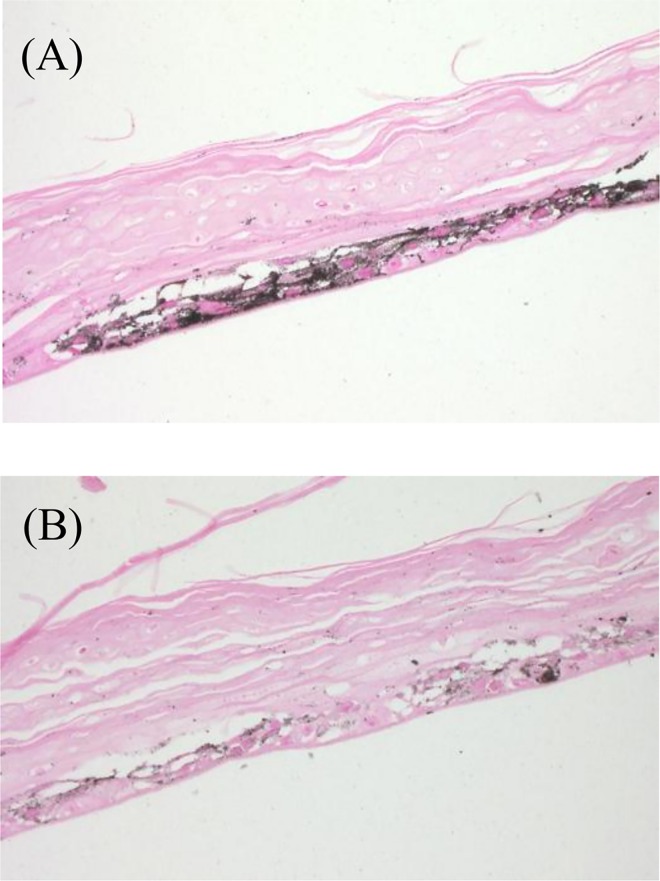
Histological changes of MEL-300-A after Fontana-Masson staining (×200). (A) O/W micelle; (B) PMCP micelle

Several reports regarding the use of MEL-300 to study the inhibitors of melanogenesis [23,24,29] show that this human skin model is useful for evaluating the effect induced by the application of whitening efficacies of cosmetic and pharmaceutical agents. It has already been shown that pigmentation of this skin tissue is assessed using the L* value-estimated skin brightness [23]. Further, tyrosinase is a key enzyme in melanin biosynthesis, and Glab isolated from licorice root extract is a powerful inhibitor of tyrosinase activity [30]. Therefore, we used MEL-300-A to assess the effect of Glab/PMCP-PM on cell pigmentation. Glab/PMCP-PM containing 0.015% (w/w) Glab-40 significantly increased the L* value **(Fig 7),** which is an index of the inhibition of melanogenesis, compared with conventional O/W micelles. Adding PQ-64 to Glab/PMCP-PM tended to enhance this effect. The cell viability rate of conventional O/W micelles, Glab/PMCP-PM, and Glab/PMCP-PM containing PQ-64 were 102.8 ± 11.8%, 104.6 ± 7.8%, and 108.5 ± 11.1%, respectively, when the skin tissue was treated, and there was no significant decrease in cell viability. These results suggest that the topical treatment of skin tissue with Glab/PMCP-PM decreased melanin synthesis without affecting cell viability. Macroscopic views of the human skin models incubated for 10 days after the application of micelles are shown in **Fig 8**. Darkening of the skin surface was clearly inhibited by the application of Glab/PMCP-PM. To visualize the melanin distribution throughout the skin tissue, Fontana-Masson staining was performed on sectioned skin. As shown in **Fig 9**, we observed that melanin production decreased with Glab/PMCP-PM treatment. These observations indicate that melanin biosynthesis in the skin was markedly decreased by Glab/PMCP-PM compared with conventional micelles.

**Fig 10:**
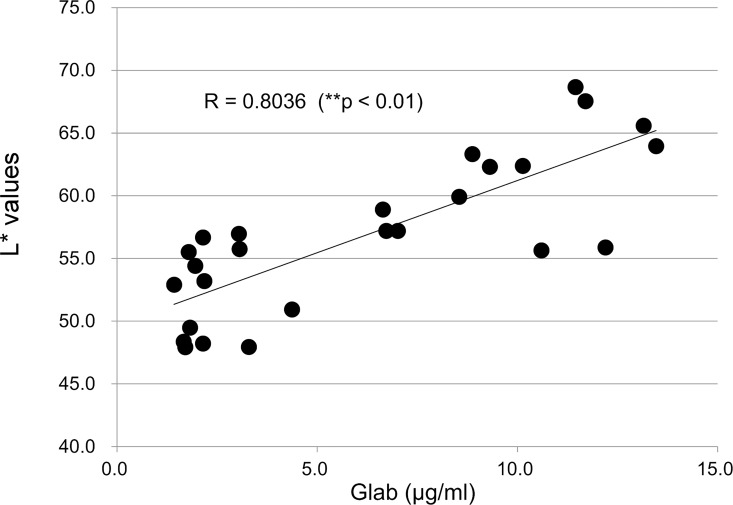
Relationship between the amount of Glab absorbed and the L* value of the skin surface of the human skin model (TESTSKIN LSE-d). The amount of Glab absorbed and the L* values on the skin surface were assayed. Correlation was assessed using Pearson’s correlation coefficient test.

We tested the effect of anionic polymer micelles formed from Pemulen TR-1 on the inhibition of melanogenesis by Glab. However, we were unable to detect a significant increase in the L* value using these conventional O/W micelles (data not shown). **Fig 10**shows the relationship between Glab absorption and inhibition of melanogenesis in a reconstituted human skin model. There was a positive correlation between the amounts of Glab absorbed in the skin model (LSE-d) and the L* values on the surface of the skin model (MEL-300-A). This result suggests that the inhibition of melanogenesis depended on enhanced penetration of Glab into the skin.

### Discussion

It is known that chitosan can be degraded into nontoxic products in vivo [17] and is safe for topical use and as an ingredient of medical devices or skin cosmetics [31]. Alginate–chitosan microcapsules [32] or chitosan nanoparticles [33] acted as a carrier for drug delivery, and chitosan, which shows biocompatibility, is useful for pharmaceutical purposes. In addition, various functional chitosan derivatives have been developed by chemical modifications of the amino group of chitosan [34]. An amphiphilic chitosan derivative with a long alkyl chain forms a polymeric micelle, which is more stable than the one formed from a low molecular weight surfactant [19]. The polymeric micellar system based on the amphiphilic chitosan acted as a carrier for hydrophobic drugs [35, 36]. However, these reports did not specify the abilities of these micelles to mediate percutaneous drug transport.

Cationic low molecular weight surfactants forming cationic micelles interact with keratin of the stratum corneum via polar interactions, thereby acting as skin penetration enhancers [37]. However, they are more destructive to skin tissues than the anionic or nonionic low molecular weight surfactants [38]. In this study, the effect of cationic, Glab-containing polymeric micelles derived from PMCP (Glab/PMCP-PM) on Glab skin penetration and melanogenesis inhibition was assessed using a three-dimensional human skin model. We have shown that the amount of Glab in the skin after the Glab/PMCP-PM application to the model was remarkably higher than that delivered by conventional O/W nanomicelles, which were formed using the nonionic low molecular weight surfactant Tween 60 **(Fig 4)**. The cationic potential of Glab/PMCP-PM is enhanced by the presence of PQ-64. Furthermore, the addition of PQ-64 to Glab/PMCP-PM tended to increase the skin penetration of Glab but had no such effect on promoting skin permeability of Glab using the conventional O/W nano-micelle procedure. Therefore, it is important to note that the effect of PQ-64 on Glab penetration to the skin model appears to be restricted to PMCP polymeric micelles. We were unable to detect a significant increase in the skin penetration of Glab using anionic polymer micelles formed from Pemulen TR-1 **(Fig 4)**. Because the human skin is negatively charged at a physiological pH and is cation permselective [22], this study suggests that Glab/PMCP-PM, which forms cationic polymer micelles having an interface rich in charged amino groups, enhances the percutaneous delivery of Glab to the human skin model. However, Glab/PMCP-PM or Glab/PMCP-PM containing PQ-64 did not increase the release of the inflammatory mediator IL-1α, which increases melanin biosynthesis **(Fig 4)**. Polymeric micelles are prepared from chitosan-grafted hydrophobic palmitoyl groups that act as a delivery system to promote the sustained release of drugs [36]. Therefore, it seems possible that Glab/PMCP-PM caused a sustained release of Glab and suppressed IL-1α release. Further experiments are required to clarify the mechanism of increased penetration of Glab mediated by Glab/PMCP-PM.

Melanin is synthesized in specialized cells called melanocytes and moves to be near keratinocytes [23]. The regulation of tyrosinase activity and/or expression plays an important role in controlling melanin production, and Glab is a powerful inhibitor of tyrosinase activity [30]. Moreover, L-tyrosine and L-DOPA, besides serving as substrates of tyrosinase and intermediates of melanogenesis, are bioregulatory agents that act as inducers and positive regulators of melanogenesis [2,3]. By the application of Glab/PMCP-PM, Glab was mainly observed in the epidermis and the amount of Glab reached a value about three times higher compared with conventional O/W nano-micelles **(Fig 5)**. **Fig 7**shows the inhibition of melanogenesis in a reconstituted human skin model by PMCP polymeric micelles containing Glab. A treatment of Glab/PMCP-PM caused a significant high melanogenesis-inhibitory effect compared to the control. Furthermore, the inhibition effect showed a tendency to increase with the addition of PQ-64 without exhibiting cytotoxicity. These results suggest that the inhibition of melanogenesis depended on the enhanced penetration of Glab into the skin, particularly the epidermis. Thus, Glab/PMCP-PM may improve the sustained release of Glab into the skin without destroying the skin barrier function, and Glab then inhibits melanin biosynthesis by melanocytes located in the basal layer of the epidermis. It appears likely that the replacement of Glab with another hydrophobic antimelanogenic agent will have a similar effect.

Skin hyperpigmentation, uneven pigmentation, age spots, melanoma, skin discoloration, and sun-damaged skin are considered unappealing and may require intensive treatment. One of the most important pigment-related skin diseases is melanoma, which is increasing in incidence in white populations and has a high mortality rate [39]. Moreover, the melanoma cells produce more melanin than the normal melanocytes in the adjacent skin [39,40]. It has recently been shown that melanogenesis shortens the overall and disease-free survivals in patients with metastatic melanoma, and the inhibition of melanogenesis appears to be a rational adjuvant approach to metastatic melanoma therapy [41]. It was also reported that Glab drastically suppressed tumor growth without statistically significant changes in the body weight of SK‑Hep-1 xenograft BALB/c nude mice [42]. From these findings, it is assumed that Glab/PMCP-PM has an effective transdermal delivery system for the treatment of skin hyperpigmentation and shows potential as an effective antimelanoma activity of percutaneous Glab as an inhibitor of melanogenesis.

Finally, it was demonstrated that a cationic polymer micelle formed from PMCP is an effective transdermal drug carrier, increasing the skin penetration of Glab into the epidermis using a human skin model. This suppressed melanin synthesis by the melanocytes present in the basal layer of the epidermis. These results further show that cationic polymer micelles generated from chitosan, which is rich in amino groups, represent a highly promising tool for the effective and safe transdermal application of antimelanogenic drugs, medicated skin care cosmetics, and medical skin clinics for treating skin hyperpigmentation.

### Conclusion

In summary, we suggest that cationic polymeric micelles constructed from PMCP are effective and safe transdermal delivery systems for the antimelanogenic agent Glab. Further studies are required to evaluate whether Glab/PMCP-PM will be clinically useful as an inhibitor of skin pigmentation
